# Redetermination of 5α-androstane-3,17-dione

**DOI:** 10.1107/S1600536810019720

**Published:** 2010-05-29

**Authors:** Jerry P. Jasinski, Ray J. Butcher, Q. N. M. Hakim Al-arique, H. S. Yathirajan, B. Narayana

**Affiliations:** a Department of Chemistry, Keene State College, 229 Main Street, Keene, NH 03435-2001, USA; b Department of Chemistry, Howard University, 525 College Street NW, Washington, DC 20059, USA; c Department of Studies in Chemistry, University of Mysore, Manasagangotri, Mysore 570 006, India; d Department of Studies in Chemistry, Mangalore University, Mangalagangotri, 574 199, India

## Abstract

The structure of the title compound, C_19_H_28_O_2_, has been redermined at 295 (2) K, with much improved precision. The structure and mol­ecular packing of the title compound was first reported by Coiro *et al.* [*Acta Cryst*. (1973). B**29**, 1404–1409] by means of potential-energy calculations. The cell parameters in this study differ considerably in space group *C*2. It is a derivative of testosterone and consists of a cyclo­penta­none ring (*A*) fused to to successive cyclo­hexane (*B* and *C*) and cyclo­hexa­none (*D*) rings. The three cyclo­hexa­none rings are in slightly distorted boat configurations and the cyclo­penta­none ring is a distorted half-chair. The crystal packing is stabilized by weak inter­molecular C—H⋯O inter­actions involving O atoms from each of the cyclo­hexa­none and cyclo­penta­none rings and H atoms from each of their respective rings.

## Related literature

For biotransformation studies, see: Fiorentino *et al.* (1991 ▶). For the previous report of this structure, see: Coiro *et al.* (1973 ▶); For related structures, see: Anthony *et al.* (1998 ▶); Jasinski *et al.* (2009 ▶); Norton *et al.* (1962 ▶); Ohrt *et al.* (1965 ▶). For bond-length data, see: Allen *et al.* (1987 ▶). For puckering parameters, see: Cremer & Pople (1975 ▶). 
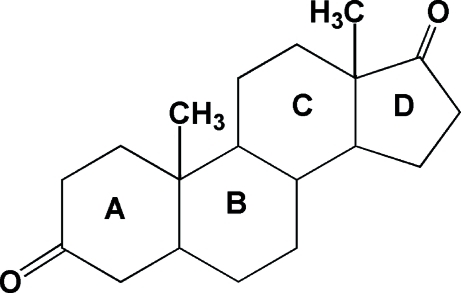


## Experimental

### 

#### Crystal data

C_19_H_28_O_2_
*M* *_r_* = 288.41
Monoclinic, 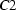

*a* = 12.7786 (6) Å
*b* = 6.7850 (4) Å
*c* = 19.6242 (10) Å
β = 106.444 (5)°
*V* = 1631.88 (15) Å^3^
*Z* = 4
Mo *K*α radiation
μ = 0.07 mm^−1^
*T* = 295 K
0.53 × 0.35 × 0.15 mm

#### Data collection

Oxford Diffraction Xcalibur Ruby Gemini diffractometer
Absorption correction: multi-scan (*CrysAlis RED*; Oxford Diffraction, 2007 ▶) *T*_min_ = 0.881, *T*_max_ = 0.989
4151 measured reflections
2082 independent reflections
1441 reflections with *I* > 2σ(*I*)
*R*_int_ = 0.020

#### Refinement

*R*[*F* ^2^ > 2σ(*F* ^2^)] = 0.040
*wR*(*F* ^2^) = 0.098
*S* = 0.96
2082 reflections
188 parameters
1 restraint
H-atom parameters constrained
Δρ_max_ = 0.16 e Å^−3^
Δρ_min_ = −0.12 e Å^−3^

### 

Data collection: *CrysAlis PRO* (Oxford Diffraction, 2007 ▶); cell refinement: *CrysAlis RED* (Oxford Diffraction, 2007 ▶); data reduction: *CrysAlis RED*; program(s) used to solve structure: *SHELXS97* (Sheldrick, 2008 ▶); program(s) used to refine structure: *SHELXL97* (Sheldrick, 2008 ▶); molecular graphics: *SHELXTL* (Sheldrick, 2008 ▶); software used to prepare material for publication: *SHELXTL* and *PLATON* (Spek, 2009 ▶).

## Supplementary Material

Crystal structure: contains datablocks global, I. DOI: 10.1107/S1600536810019720/om2341sup1.cif

Structure factors: contains datablocks I. DOI: 10.1107/S1600536810019720/om2341Isup2.hkl

Additional supplementary materials:  crystallographic information; 3D view; checkCIF report

## Figures and Tables

**Table 1:** Hydrogen-bond geometry (Å, °)

*D*—H⋯*A*	*D*—H	H⋯*A*	*D*⋯*A*	*D*—H⋯*A*
C16—H16*A*⋯O2^i^	0.97	2.61	3.247 (3)	123
C5—H5*A*⋯O1^ii^	0.97	2.61	3.335 (3)	131

Symmetry codes: (i) 
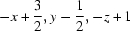
; (ii) 
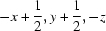
.

**Fig. 1.**
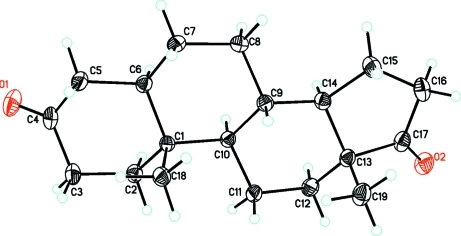
Molecular structure of C19H28O2, showing the atom labeling scheme and 50% probability displacement ellipsoids.

**Fig. 2.**
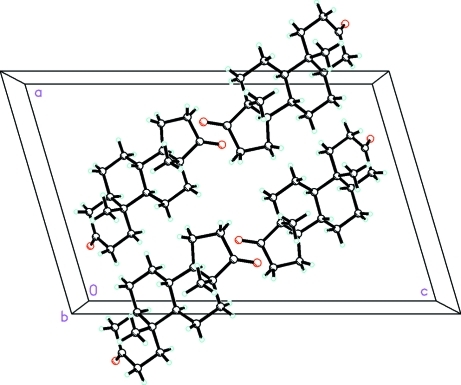
Packing diagram of the title compound viewed down the b axis.

## supplementary crystallographic information

### Comment

The title compound, C_19_H_28_O_2_ (systematic iupac name: (8*R*,9S,10*R*,13S,14*S*)-10,13-Dimethyl-1,2,4,5,6,7,8,9,11,12,14,15,16- tridecahydrocyclopenta[*a*]phenanthrane-3,17-dione or dodecahydro-10,13-dimethyl-2*H*-cyclopenta[*a*]phenanthrene-3,17(4*H*,14H)-dione) is a derivative of testosterone. The crystal structure of the title compound (CSD code: ANDION10) was first reported by Coiro *et al.* (1973) with cell parameters of a = 12.700 (20); b = 6.190 (10); c = 21.340 (30) Å; c = 91.27 (10)° and an *R*-factor of 12% at T = 295 K. These values coincide with those reported earlier (Ohrt *et al.*, 1965). How ever in the present investigations, the cell parameters are a = 12.7786 (6); b = 6.7850 (4); c = 19.6242 (10) Å and β = 106.444 (5)° with an *R*-factor of 3.86% at 295 K. The crystal structure of 5β-androstane-3,17-dione is already reported (Norton *et al.*, 1962; Anthony *et al.*, 1998). The biotransformation of 5α-androstane-3,17-dione by microalgal cultures is reported (Fiorentino *et al.*, 1991). We have recently reported the crystal structure of 3-oxo-4-aza-5-alpha-androstone-17*b*-*tert* -butyl carboxamide (Jasinski *et al.*, 2009). In view of the importance of steroids and taking into account the importance of the title compound, this paper reports the redetermination of its crystal structure with an improved precision.

The title compound, C_19_H_28_O_2_, consists of a cyclopentanone ring (A) fused to to successive cyclohexane (B & C) and cyclohexanone (D) rings. The three cyclohexanone rings are in slightly distorted chair configurations with Cremer & Pople (1975) puckering parameters Q, θ and φ of 0.530 (2) Å, 9.5 (2)° & 3.5 (13)°, for A, 0.5778 (19) Å, 5.77 (19)° & 344.7 (19)°, for B, and 0.5697 (19) Å, 173.01 (19)° & 65.5 (16)°,for C, respectively (Fig. 1). For an ideal chair θ has a value of 0 or 180°. The cyclopentanone ring is a distorted half-chair, phi(2) = 22.5 (3)°. For an ideal half-chair, phi(2) = k *x* 36 + 18. The crystal packing is stabilized by weak intermolecular C–H···O interactions between a hydrogen atom from the cyclohexanone ring (H5A) with an oxygen atom from a nearby cyclohexanone ring (O1) and between a hydrogen atom from the cyclopentanone ring (H16A) with an oxygen atom from a nearby cyclopentanone ring (O2), respectively (Fig. 2, Table 1).

### Experimental

The title compound was obtained as a gift sample from *R. L*. Fine Chem., Bangalore, India. The compound was used without further purification. X-ray quality crystals were obtained from slow evaporation of acetone solution (m.p.: 373–375 K).

### Refinement

All of the H atoms were placed in their calculated positions and then refined using the riding model with C—H = 0.96–0.98 Å, and with *U*_iso_(H) = 1.18–1.50*U*_eq_(C).

### Crystal data

**Table:**

C_19_H_28_O_2_	*F*(000) = 632
*M**_r_* = 288.41	*D*_x_ = 1.174 Mg m^−^^3^
Monoclinic, *C*2	Mo *K*α radiation, λ = 0.71073 Å
Hall symbol: C 2y	Cell parameters from 1842 reflections
*a* = 12.7786 (6) Å	θ = 5.1–29.4°
*b* = 6.7850 (4) Å	µ = 0.07 mm^−^^1^
*c* = 19.6242 (10) Å	*T* = 295 K
β = 106.444 (5)°	Plate, colorless
*V* = 1631.88 (15) Å^3^	0.53 × 0.35 × 0.15 mm
*Z* = 4	

### Data collection

**Table:**

Oxford Diffraction Xcalibur Ruby Gemini diffractometer	2082 independent reflections
Radiation source: Enhance (Mo) X-ray Source	1441 reflections with *I* > 2σ(*I*)
graphite	*R*_int_ = 0.020
Detector resolution: 10.5081 pixels mm^-1^	θ_max_ = 29.5°, θ_min_ = 5.2°
ω scans	*h* = −17→16
Absorption correction: multi-scan (*CrysAlis RED*; Oxford Diffraction, 2007)	*k* = −7→9
*T*_min_ = 0.881, *T*_max_ = 0.989	*l* = −22→24
4151 measured reflections	

### Refinement

**Table:**

Refinement on *F*^2^	Primary atom site location: structure-invariant direct methods
Least-squares matrix: full	Secondary atom site location: difference Fourier map
*R*[*F*^2^ > 2σ(*F*^2^)] = 0.040	Hydrogen site location: inferred from neighbouring sites
*wR*(*F*^2^) = 0.098	H-atom parameters constrained
*S* = 0.96	*w* = 1/[σ^2^(*F*_o_^2^) + (0.0558*P*)^2^] where *P* = (*F*_o_^2^ + 2*F*_c_^2^)/3
2082 reflections	(Δ/σ)_max_ < 0.001
188 parameters	Δρ_max_ = 0.16 e Å^−^^3^
1 restraint	Δρ_min_ = −0.12 e Å^−^^3^

### Special details

**Table:**

Experimental. In the absence of anolmalous scattering effects Friedel opposites were merged.
Geometry. All e.s.d.'s (except the e.s.d. in the dihedral angle between two l.s. planes) are estimated using the full covariance matrix. The cell e.s.d.'s are taken into account individually in the estimation of e.s.d.'s in distances, angles and torsion angles; correlations between e.s.d.'s in cell parameters are only used when they are defined by crystal symmetry. An approximate (isotropic) treatment of cell e.s.d.'s is used for estimating e.s.d.'s involving l.s. planes.
Refinement. Refinement of *F*^2^ against ALL reflections. The weighted *R*-factor *wR* and goodness of fit *S* are based on *F*^2^, conventional *R*-factors *R* are based on *F*, with *F* set to zero for negative *F*^2^. The threshold expression of *F*^2^ > σ(*F*^2^) is used only for calculating *R*-factors(gt) *etc*. and is not relevant to the choice of reflections for refinement. *R*-factors based on *F*^2^ are statistically about twice as large as those based on *F*, and *R*- factors based on ALL data will be even larger.

### Fractional atomic coordinates and isotropic or equivalent isotropic displacement parameters (Å^2^)

**Table:**

	*x*	*y*	*z*	*U*_iso_*/*U*_eq_	
O1	0.24755 (15)	−0.2050 (4)	0.04123 (10)	0.0831 (6)	
O2	0.70862 (13)	0.4649 (3)	0.51542 (8)	0.0661 (5)	
C1	0.42695 (14)	0.2013 (3)	0.18578 (9)	0.0343 (5)	
C2	0.31200 (15)	0.1301 (4)	0.18422 (10)	0.0463 (6)	
H2A	0.3186	0.0144	0.2141	0.056*	
H2B	0.2764	0.2322	0.2042	0.056*	
C3	0.23997 (15)	0.0794 (4)	0.10955 (11)	0.0517 (6)	
H3A	0.1721	0.0225	0.1131	0.062*	
H3B	0.2226	0.1994	0.0818	0.062*	
C4	0.29440 (18)	−0.0622 (4)	0.07223 (11)	0.0524 (6)	
C5	0.40981 (16)	−0.0152 (4)	0.07567 (10)	0.0499 (6)	
H5A	0.4108	0.0940	0.0439	0.060*	
H5B	0.4423	−0.1282	0.0592	0.060*	
C6	0.47906 (15)	0.0392 (3)	0.15109 (10)	0.0409 (5)	
H6A	0.4821	−0.0793	0.1801	0.049*	
C7	0.59602 (15)	0.0857 (4)	0.15289 (10)	0.0474 (6)	
H7A	0.5971	0.2011	0.1239	0.057*	
H7B	0.6262	−0.0240	0.1329	0.057*	
C8	0.66634 (15)	0.1239 (4)	0.22867 (10)	0.0470 (6)	
H8A	0.7380	0.1673	0.2273	0.056*	
H8B	0.6754	0.0013	0.2551	0.056*	
C9	0.61800 (14)	0.2786 (3)	0.26770 (10)	0.0379 (5)	
H9A	0.6179	0.4062	0.2442	0.045*	
C10	0.49888 (15)	0.2242 (3)	0.26409 (9)	0.0354 (5)	
H10A	0.5024	0.0928	0.2853	0.043*	
C11	0.44999 (15)	0.3593 (4)	0.31000 (10)	0.0453 (6)	
H11A	0.4379	0.4889	0.2883	0.054*	
H11B	0.3797	0.3071	0.3108	0.054*	
C12	0.52271 (16)	0.3798 (4)	0.38663 (10)	0.0494 (6)	
H12A	0.4908	0.4743	0.4120	0.059*	
H12B	0.5279	0.2539	0.4108	0.059*	
C13	0.63549 (15)	0.4483 (3)	0.38639 (9)	0.0414 (5)	
C14	0.68430 (14)	0.2984 (4)	0.34512 (10)	0.0407 (5)	
H14A	0.6812	0.1698	0.3672	0.049*	
C15	0.80574 (15)	0.3562 (4)	0.36433 (11)	0.0566 (7)	
H15A	0.8504	0.2457	0.3583	0.068*	
H15B	0.8172	0.4654	0.3354	0.068*	
C16	0.83153 (18)	0.4161 (5)	0.44290 (11)	0.0575 (7)	
H16A	0.8722	0.3128	0.4732	0.069*	
H16B	0.8744	0.5362	0.4517	0.069*	
C17	0.72336 (18)	0.4479 (4)	0.45736 (11)	0.0492 (6)	
C18	0.41689 (15)	0.3991 (3)	0.14581 (8)	0.0457 (6)	
H18A	0.3770	0.3796	0.0969	0.069*	
H18B	0.4884	0.4484	0.1486	0.069*	
H18C	0.3790	0.4923	0.1670	0.069*	
C19	0.63290 (18)	0.6613 (3)	0.35832 (8)	0.0579 (6)	
H19A	0.6002	0.7465	0.3856	0.087*	
H19B	0.5909	0.6651	0.3093	0.087*	
H19C	0.7060	0.7046	0.3626	0.087*	

### Atomic displacement parameters (Å^2^)

**Table:**

	*U*^11^	*U*^22^	*U*^33^	*U*^12^	*U*^13^	*U*^23^
O1	0.0922 (13)	0.0866 (15)	0.0664 (12)	−0.0309 (13)	0.0158 (9)	−0.0327 (12)
O2	0.0834 (11)	0.0740 (13)	0.0335 (8)	0.0045 (10)	0.0042 (7)	−0.0029 (9)
C1	0.0359 (9)	0.0376 (12)	0.0287 (10)	0.0022 (9)	0.0080 (7)	0.0006 (9)
C2	0.0453 (11)	0.0547 (16)	0.0382 (12)	−0.0025 (11)	0.0108 (9)	−0.0039 (11)
C3	0.0444 (11)	0.0677 (18)	0.0398 (12)	−0.0127 (12)	0.0065 (9)	−0.0080 (12)
C4	0.0608 (13)	0.0567 (16)	0.0332 (11)	−0.0103 (14)	0.0029 (9)	−0.0024 (12)
C5	0.0587 (13)	0.0544 (16)	0.0340 (11)	0.0040 (12)	0.0090 (9)	−0.0069 (11)
C6	0.0471 (11)	0.0378 (12)	0.0354 (11)	0.0026 (10)	0.0079 (8)	−0.0010 (10)
C7	0.0475 (11)	0.0560 (16)	0.0400 (12)	0.0092 (12)	0.0148 (9)	−0.0080 (11)
C8	0.0383 (10)	0.0544 (15)	0.0481 (13)	0.0069 (11)	0.0117 (9)	−0.0061 (11)
C9	0.0395 (10)	0.0398 (13)	0.0329 (10)	0.0032 (10)	0.0077 (8)	0.0021 (9)
C10	0.0400 (10)	0.0353 (12)	0.0293 (10)	0.0012 (9)	0.0071 (8)	0.0014 (9)
C11	0.0417 (10)	0.0557 (15)	0.0386 (11)	0.0009 (11)	0.0115 (8)	−0.0092 (11)
C12	0.0562 (12)	0.0582 (16)	0.0351 (11)	0.0015 (12)	0.0150 (9)	−0.0058 (11)
C13	0.0488 (11)	0.0417 (13)	0.0293 (10)	0.0016 (11)	0.0041 (8)	−0.0008 (10)
C14	0.0426 (10)	0.0389 (12)	0.0371 (11)	0.0008 (11)	0.0056 (8)	0.0035 (10)
C15	0.0448 (11)	0.073 (2)	0.0465 (12)	−0.0008 (12)	0.0040 (9)	0.0025 (13)
C16	0.0530 (12)	0.0652 (18)	0.0446 (12)	−0.0114 (13)	−0.0020 (9)	0.0021 (13)
C17	0.0635 (14)	0.0407 (14)	0.0354 (12)	−0.0027 (12)	0.0012 (9)	0.0036 (11)
C18	0.0497 (11)	0.0443 (14)	0.0389 (11)	0.0036 (11)	0.0055 (8)	0.0035 (11)
C19	0.0737 (15)	0.0435 (14)	0.0460 (14)	−0.0014 (14)	0.0000 (11)	−0.0005 (12)

### Geometric parameters (Å, °)

**Table:**

O1—C4	1.209 (3)	C9—H9A	0.9800
O2—C17	1.212 (2)	C10—C11	1.536 (3)
C1—C2	1.538 (3)	C10—H10A	0.9800
C1—C6	1.541 (3)	C11—C12	1.534 (3)
C1—C18	1.541 (3)	C11—H11A	0.9700
C1—C10	1.559 (2)	C11—H11B	0.9700
C2—C3	1.533 (3)	C12—C13	1.515 (3)
C2—H2A	0.9700	C12—H12A	0.9700
C2—H2B	0.9700	C12—H12B	0.9700
C3—C4	1.494 (3)	C13—C17	1.521 (3)
C3—H3A	0.9700	C13—C14	1.538 (3)
C3—H3B	0.9700	C13—C19	1.544
C4—C5	1.492 (3)	C14—C15	1.540 (3)
C5—C6	1.539 (3)	C14—H14A	0.9800
C5—H5A	0.9700	C15—C16	1.538 (3)
C5—H5B	0.9700	C15—H15A	0.9700
C6—C7	1.518 (3)	C15—H15B	0.9700
C6—H6A	0.9800	C16—C17	1.503 (3)
C7—C8	1.526 (3)	C16—H16A	0.9700
C7—H7A	0.9700	C16—H16B	0.9700
C7—H7B	0.9700	C18—H18A	0.9600
C8—C9	1.529 (3)	C18—H18B	0.9600
C8—H8A	0.9700	C18—H18C	0.9600
C8—H8B	0.9700	C19—H19A	0.9600
C9—C14	1.522 (2)	C19—H19B	0.9600
C9—C10	1.548 (2)	C19—H19C	0.9600
			
C2—C1—C6	107.26 (17)	C11—C10—H10A	105.7
C2—C1—C18	108.76 (16)	C9—C10—H10A	105.7
C6—C1—C18	112.39 (15)	C1—C10—H10A	105.7
C2—C1—C10	110.12 (14)	C12—C11—C10	113.26 (16)
C6—C1—C10	107.39 (15)	C12—C11—H11A	108.9
C18—C1—C10	110.84 (16)	C10—C11—H11A	108.9
C3—C2—C1	113.65 (15)	C12—C11—H11B	108.9
C3—C2—H2A	108.8	C10—C11—H11B	108.9
C1—C2—H2A	108.8	H11A—C11—H11B	107.7
C3—C2—H2B	108.8	C13—C12—C11	109.73 (15)
C1—C2—H2B	108.8	C13—C12—H12A	109.7
H2A—C2—H2B	107.7	C11—C12—H12A	109.7
C4—C3—C2	112.10 (18)	C13—C12—H12B	109.7
C4—C3—H3A	109.2	C11—C12—H12B	109.7
C2—C3—H3A	109.2	H12A—C12—H12B	108.2
C4—C3—H3B	109.2	C12—C13—C17	116.93 (17)
C2—C3—H3B	109.2	C12—C13—C14	109.07 (18)
H3A—C3—H3B	107.9	C17—C13—C14	100.18 (16)
O1—C4—C5	122.5 (2)	C12—C13—C19	111.32
O1—C4—C3	122.0 (2)	C17—C13—C19	105.30
C5—C4—C3	115.6 (2)	C14—C13—C19	113.70
C4—C5—C6	112.76 (16)	C9—C14—C13	112.95 (16)
C4—C5—H5A	109.0	C9—C14—C15	120.29 (16)
C6—C5—H5A	109.0	C13—C14—C15	103.50 (18)
C4—C5—H5B	109.0	C9—C14—H14A	106.4
C6—C5—H5B	109.0	C13—C14—H14A	106.4
H5A—C5—H5B	107.8	C15—C14—H14A	106.4
C7—C6—C5	111.51 (16)	C16—C15—C14	103.17 (17)
C7—C6—C1	112.77 (17)	C16—C15—H15A	111.1
C5—C6—C1	113.16 (16)	C14—C15—H15A	111.1
C7—C6—H6A	106.3	C16—C15—H15B	111.1
C5—C6—H6A	106.3	C14—C15—H15B	111.1
C1—C6—H6A	106.3	H15A—C15—H15B	109.1
C6—C7—C8	111.24 (16)	C17—C16—C15	106.24 (17)
C6—C7—H7A	109.4	C17—C16—H16A	110.5
C8—C7—H7A	109.4	C15—C16—H16A	110.5
C6—C7—H7B	109.4	C17—C16—H16B	110.5
C8—C7—H7B	109.4	C15—C16—H16B	110.5
H7A—C7—H7B	108.0	H16A—C16—H16B	108.7
C7—C8—C9	113.24 (17)	O2—C17—C16	125.95 (19)
C7—C8—H8A	108.9	O2—C17—C13	126.1 (2)
C9—C8—H8A	108.9	C16—C17—C13	107.90 (17)
C7—C8—H8B	108.9	C1—C18—H18A	109.5
C9—C8—H8B	108.9	C1—C18—H18B	109.5
H8A—C8—H8B	107.7	H18A—C18—H18B	109.5
C14—C9—C8	111.77 (16)	C1—C18—H18C	109.5
C14—C9—C10	109.20 (14)	H18A—C18—H18C	109.5
C8—C9—C10	110.16 (17)	H18B—C18—H18C	109.5
C14—C9—H9A	108.5	C13—C19—H19A	109.5
C8—C9—H9A	108.5	C13—C19—H19B	109.4
C10—C9—H9A	108.5	H19A—C19—H19B	109.5
C11—C10—C9	112.88 (16)	C13—C19—H19C	109.5
C11—C10—C1	114.42 (15)	H19A—C19—H19C	109.5
C9—C10—C1	111.49 (14)	H19B—C19—H19C	109.5
			
C6—C1—C2—C3	−56.4 (3)	C6—C1—C10—C9	58.7 (2)
C18—C1—C2—C3	65.4 (2)	C18—C1—C10—C9	−64.4 (2)
C10—C1—C2—C3	−173.0 (2)	C9—C10—C11—C12	−50.8 (2)
C1—C2—C3—C4	52.7 (3)	C1—C10—C11—C12	−179.73 (17)
C2—C3—C4—O1	133.4 (2)	C10—C11—C12—C13	54.9 (3)
C2—C3—C4—C5	−46.8 (3)	C11—C12—C13—C17	−171.28 (19)
O1—C4—C5—C6	−133.6 (2)	C11—C12—C13—C14	−58.6 (2)
C3—C4—C5—C6	46.6 (3)	C11—C12—C13—C19	67.6
C4—C5—C6—C7	179.9 (2)	C8—C9—C14—C13	−178.12 (18)
C4—C5—C6—C1	−51.8 (3)	C10—C9—C14—C13	−56.0 (2)
C2—C1—C6—C7	−176.75 (16)	C8—C9—C14—C15	59.1 (3)
C18—C1—C6—C7	63.8 (2)	C10—C9—C14—C15	−178.7 (2)
C10—C1—C6—C7	−58.4 (2)	C12—C13—C14—C9	61.7 (2)
C2—C1—C6—C5	55.5 (2)	C17—C13—C14—C9	−175.01 (17)
C18—C1—C6—C5	−64.0 (2)	C19—C13—C14—C9	−63.2
C10—C1—C6—C5	173.87 (17)	C12—C13—C14—C15	−166.62 (17)
C5—C6—C7—C8	−175.6 (2)	C17—C13—C14—C15	−43.3 (2)
C1—C6—C7—C8	55.8 (2)	C19—C13—C14—C15	68.5
C6—C7—C8—C9	−52.4 (3)	C9—C14—C15—C16	164.6 (2)
C7—C8—C9—C14	174.44 (19)	C13—C14—C15—C16	37.4 (2)
C7—C8—C9—C10	52.8 (2)	C14—C15—C16—C17	−16.2 (3)
C14—C9—C10—C11	49.8 (2)	C15—C16—C17—O2	167.1 (3)
C8—C9—C10—C11	172.87 (17)	C15—C16—C17—C13	−11.1 (3)
C14—C9—C10—C1	−179.82 (18)	C12—C13—C17—O2	−27.0 (3)
C8—C9—C10—C1	−56.7 (2)	C14—C13—C17—O2	−144.6 (3)
C2—C1—C10—C11	−55.2 (2)	C19—C13—C17—O2	97.2
C6—C1—C10—C11	−171.69 (18)	C12—C13—C17—C16	151.2 (2)
C18—C1—C10—C11	65.2 (2)	C14—C13—C17—C16	33.6 (2)
C2—C1—C10—C9	175.18 (19)	C19—C13—C17—C16	−84.6

### Hydrogen-bond geometry (Å, °)

**Table:**

*D*—H···*A*	*D*—H	H···*A*	*D*···*A*	*D*—H···*A*
C16—H16A···O2^i^	0.97	2.61	3.247 (3)	123
C5—H5A···O1^ii^	0.97	2.61	3.335 (3)	131

Symmetry codes: (i) −*x*+3/2, *y*−1/2, −*z*+1; (ii) −*x*+1/2, *y*+1/2, −*z*.
